# Mucoromycotina Fine Root Endophyte Fungi Form Nutritional Mutualisms with Vascular Plants^1^

**DOI:** 10.1104/pp.19.00729

**Published:** 2019-07-29

**Authors:** Grace A. Hoysted, Alison S. Jacob, Jill Kowal, Philipp Giesemann, Martin I. Bidartondo, Jeffrey G. Duckett, Gerhard Gebauer, William R. Rimington, Sebastian Schornack, Silvia Pressel, Katie J. Field

**Affiliations:** a Centre for Plant Sciences, Faculty of Biological Sciences, University of Leeds, Leeds, LS2 9JT, United Kingdom; b Comparative Plant & Fungal Biology, Royal Botanic Gardens, Kew, Richmond TW9 3DS, United Kingdom; c Department of Life Sciences, Imperial College London, London, SW7 2AZ, United Kingdom; d Department of Life Sciences, Natural History Museum, London SW7 5BD, United Kingdom; e Laboratory of Isotope Biogeochemistry, Bayreuth Center of Ecology and Environmental Research, University of Bayreuth, 95440 Bayreuth, Germany; f Sainsbury Laboratory, University of Cambridge, Cambridge, CB2 1LR, United Kingdom

## Abstract

Fungi and plants have engaged in intimate symbioses that are globally widespread and have driven terrestrial biogeochemical processes since plant terrestrialization >500 million years ago. Recently, hitherto unknown nutritional mutualisms involving ancient lineages of fungi and nonvascular plants have been discovered, although their extent and functional significance in vascular plants remain uncertain. Here, we provide evidence of carbon-for-nitrogen exchange between an early-diverging vascular plant (*Lycopodiella inundata*) and Mucoromycotina (Endogonales) fine root endophyte fungi. Furthermore, we demonstrate that the same fungal symbionts colonize neighboring nonvascular and flowering plants. These findings fundamentally change our understanding of the physiology, interrelationships, and ecology of underground plant–fungal symbioses in modern terrestrial ecosystems by revealing the nutritional role of Mucoromycotina fungal symbionts in vascular plants.

## Abstract (precis)

Mucoromycotina fine root endophyte fungi transfer soil nutrients, notably N, to lycophyte host plants in exchange for photosynthetically fixed C and colonize diverse neighboring vascular plants.

Plant terrestrialization >500 million years ago (Morris et al., 2018) was facilitated by the formation of mutualistic symbioses with fungi, through which the earliest plants gained access to mineral nutrients in exchange for photosynthetically fixed carbon (C). It was long hypothesized that this ancient mycorrhiza-like symbiosis was closely related to, and subsequently evolved into, widespread modern-day arbuscular mycorrhizas (AM) formed with plant roots by Glomeromycotina fungi (Pirozynski and Malloch, 1975; Redecker et al., 2000). However, recent molecular, cytological, physiological, and paleobotanical evidence has strongly indicated that early fungal associates were likely to be more diverse than has previously been assumed (Bidartondo et al., 2011; Field et al., 2015a, 2015b). Members of the earliest diverging clade of an ancient land plant lineage, Haplomitriopsida liverworts, are now known to form mycorrhiza-like associations with Mucoromycotina fungi (Bidartondo et al., 2011; Field et al., 2012, 2015b), which also colonize other early diverging plant lineages, namely hornworts, lycophytes, and ferns, sometimes co-occurring with Glomeromycotina fungi in the same plant host (Desirò et al., 2013; Rimington et al., 2015). Mucoromycotina represents an ancient fungal lineage considered to branch earlier than, or as a sister to, the Glomeromycotina AM fungi (James et al., 2006; Lin et al., 2014). The recent identification of Mucoromycotina in a range of modern nonvascular plants (Bidartondo et al., 2011) and plant fossils (Krings et al., 2007; Strullu-Derrien et al., 2014) supports the idea that the colonization of Earth’s land masses by plants was facilitated not only by Glomeromycotina AM, but also by Mucoromycotina fungal symbionts (Field et al., 2015a). The latest discoveries of putative Mucoromycotina fungi in vascular land plants (Rimington et al., 2015, 2016; Orchard et al., 2017a) indicate that root symbiotic versatility and diversity (Hoysted et al., 2018) has been grossly underestimated across extant plants.

Although Mucoromycotina fungal symbioses in nonvascular plants have received the most attention to date, there are now several reports of their occurrence in vascular plants (Rimington et al., 2015, 2016; Orchard et al., 2017a, 2017b; Hoysted et al., 2018). It has been suggested that the globally widespread, arbuscule-forming fine root endophytes (FREs) classified as *Glomus tenue* (or, more recently, *Planticonsortium tenue*; Walker et al., 2018), which occur across a wide range of vascular plant groups (Rimington et al., 2015; Orchard et al., 2017b), are closely related to the Mucoromycotina fungal symbionts of nonvascular plants (Field and Pressel, 2018; Hoysted et al., 2018). If true, there could be major ramifications for our understanding of the past and present diversity and function of plant–fungal nutritional symbioses (Field and Pressel, 2018), suggesting Mucoromycotina fungal symbiosis is not limited to ancient plant lineages but is in fact widespread throughout extant land plants. However, it remains unclear whether the putative Mucoromycotina FREs detected in vascular plants to date are comparable in terms of function and identity to the mutualistic Mucoromycotina fungal symbionts detected in nonvascular plants.

As lycophytes are considered to be the earliest divergent extant vascular plant lineage (Kenrick and Crane, 1997), the discovery of non-Glomeromycotina fungal associates in lycophyte roots and gametophytes is particularly important. For over 100 years, fungal associations in lycophytes have been thought of as being AM-like but with unique “lycopodioid” features (Duckett and Ligrone, 1992; Schmid and Oberwinkler, 1993). However, global analysis of fungal associates in 20 lycophytes (Rimington et al., 2016) has now shown their colonization is broadly similar to that of hornworts (Desirò et al., 2013), with many species forming single and/or dual associations with both Glomeromycotina AM fungi and Mucoromycotina FRE fungi (Rimington et al., 2016). Remarkably, every sample of *Lycopodiella inundata—*a species found in wet habitats across the northern Hemisphere—examined so far appears colonized exclusively by Mucoromycotina FRE fungi (Rimington et al., 2016). The fundamental obstacle to studying function of Mucoromycotina FREs has been finding any plants that are not co-colonized by coarse root endophytes (i.e. Glomeromycotina AM fungi). In fact, so far there is no evidence of nutritional mutualism between any vascular plant and Mucoromycotina FREs (Hoysted et al., 2018). Therefore, *L*. *inundata* provides a unique and important opportunity to dissect the symbiotic function of FREs in a vascular plant.

Here, we investigate the function, cytology, and occurrence of the fungal associates of *L*. *inundata* (Fig. 1). We use a combination of radio- and stable isotope tracers (Supplemental Figs. S1 and S2) to test physiology and functioning of vascular plant-Mucoromycotina fungal symbioses, detailed cytological analyses to characterize morphology and colonization patterns of Mucoromycotina fungi, and molecular techniques to identify the fungal associates of a range of vascular plants (including those used in our experiments) across several field sites. Furthermore, we used natural abundance δ^13^C and δ^15^N signatures to test under field conditions whether Mucoromycotina fungal association affects the direction of carbon and nitrogen fluxes between plants and associated fungi (Gebauer and Meyer, 2003). Specifically, we address the following questions:

**Figure 1.**
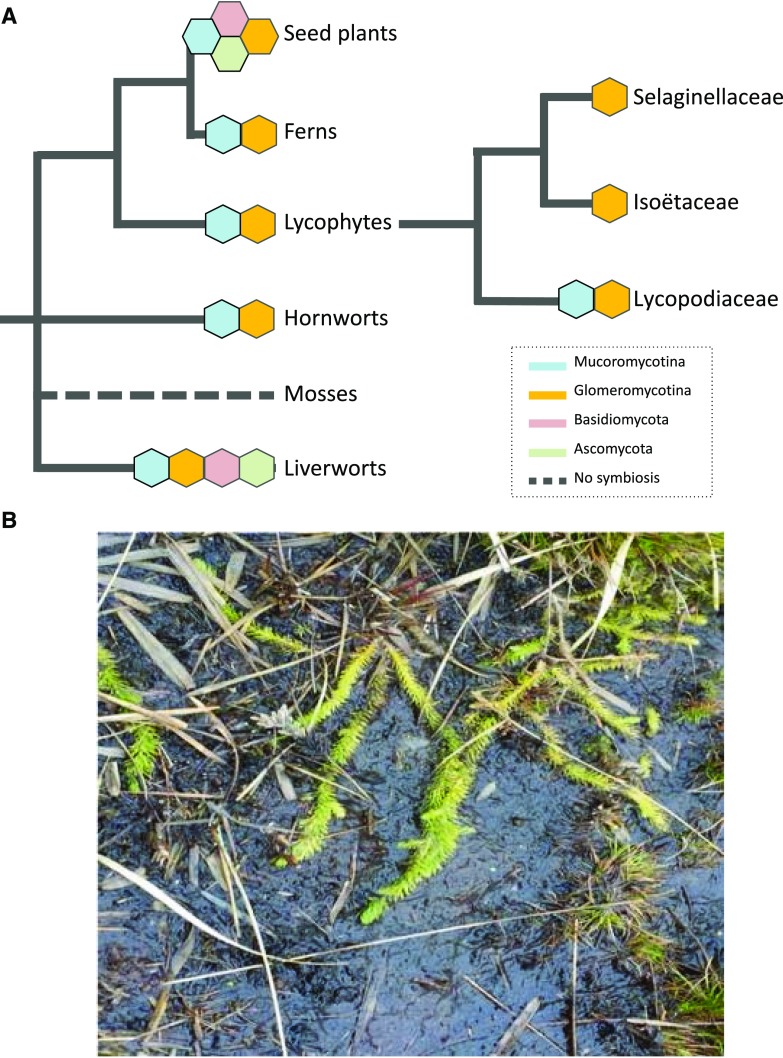
Land plant phylogeny and species used in this study. A, Land plant phylogeny showing key nodes alongside commonly associated fungal symbionts (Duckett and Ligrone, 1992; Duckett et al., 2006; James et al., 2006; Bidartondo et al., 2011). B, *L*. *inundata* at Thursley Common, Surrey, United Kingdom, June 2017.

1. What is the function of Mucoromycotina fungal associations in lycophytes in terms of carbon-for-nutrient exchange?
2. Are there characteristic cytological signatures or features of Mucoromycotina fungal associations in *L*. *inundata* and other vascular plants?
3. Do Mucoromycotina fungal symbionts of *L*. *inundata* co-occur in neighboring angiosperm roots and nonvascular plants?

## RESULTS

### Mucoromycotina–*L*. *inundata* Symbioses Are Nutritional Mutualisms

**Figure 2.**
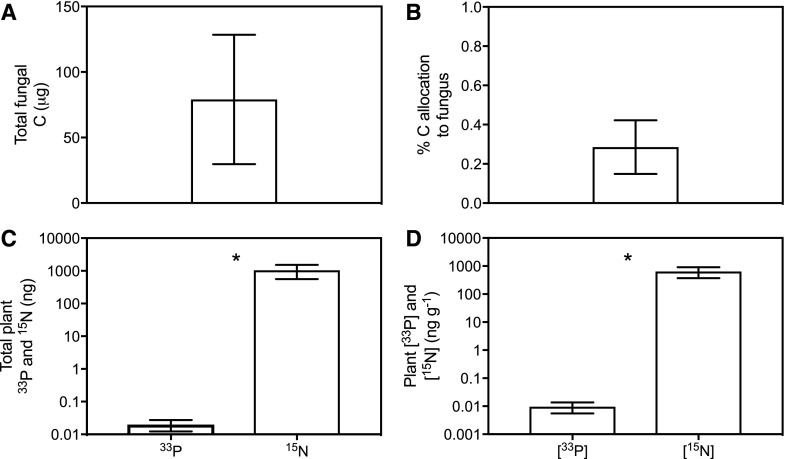
Carbon-for-nutrient exchange between *L*. *inundata* and Mucoromycotina fine root endophyte fungi. A, Total plant-fixed carbon transferred to Mucoromycotina FRE fungi by *L*. *inundata*. B, Percent allocation of plant-fixed carbon to Mucoromycotina FRE fungi. C and D, Total plant tissue phosphorus (^33^P) and nitrogen (^15^N) content in nanograms (C) and tissue concentration (ng g^−1^) of fungal-acquired ^33^P and ^15^N in *L*. *inundata* tissue (D). In (A) and (B), *n* = 20; in (C) and (D), *n* = 10 (*n* indicates the number of biological replicates used during carbon-for-nutrient exchange experiments). Experiments were carried out three times. Asterisk (*) indicates where *P* < 0.05, Student’s *t* test. Error bars = means ± se.

Isotope tracing experiments conducted in controlled environment chambers confirmed carbon was transferred from field-collected *L*. *inundata* to extraradical hyphae of symbiotic Mucoromycotina FRE fungi (Fig. 2). Mucoromycotina fungal symbiont identity was confirmed using sequencing of the fungal 18S ribosomal RNA (rRNA) gene with the broad specificity fungal primer set NS1/EF3 and a semi-nested approach with Mucoromycotina- and Glomeromycotina-specific primers described in Desirò et al. (2013); Supplemental Fig. S3). Plants transferred an average of 79 μg (±49.3 se) of recent photosynthate to the external fungal hyphal mycelium within the microcosm during the labeling period (Fig. 2A). This represents 0.28% (±0.14 se) of the total amount of carbon that was fixed during the labeling period by *L*. *inundata* (Fig. 2B).

Mucoromycotina fungi within the experimental microcosms transferred between 3% and 9% of the supplied ^33^P tracer and 0.6% to 1% of the supplied ^15^N tracer to their plant hosts during the isotope labeling period (Fig. 2, C and D). Mucoromycotina FREs transferred significantly more ^15^N than ^33^P to *L*. *inundata* in terms of both absolute quantities (Fig. 2C, *P* = 0.05; Student’s *t* test) and when normalized to plant biomass (Fig. 2D, *P* = 0.03, Student’s *t* test).

To test the potential nutritional role of Mucoromycotina fungi in *L*. *inundata* in the field, we analyzed the natural abundance ^13^C and ^15^N stable isotope signatures of leaves and roots of *L*. *inundata* and *Juncus bulbosus*, both of which were shown to host Mucoromycotina FREs in their roots (Supplemental Figs. S4–S9). In addition, five plant species representing three different types of mycorrhizal associations were sampled to serve as reference plants: two ericoid mycorrhizal species (*Erica tetralix*, collected on six plots; *Calluna vulgaris*, collected on three plots), two ectomycorrhizal species (*Pinus sylvestris* and *Betula pendula* seedlings, both from one plot), and one arbuscular mycorrhizal species (*Molinia caerulea* from six plots).

**Figure 3.**
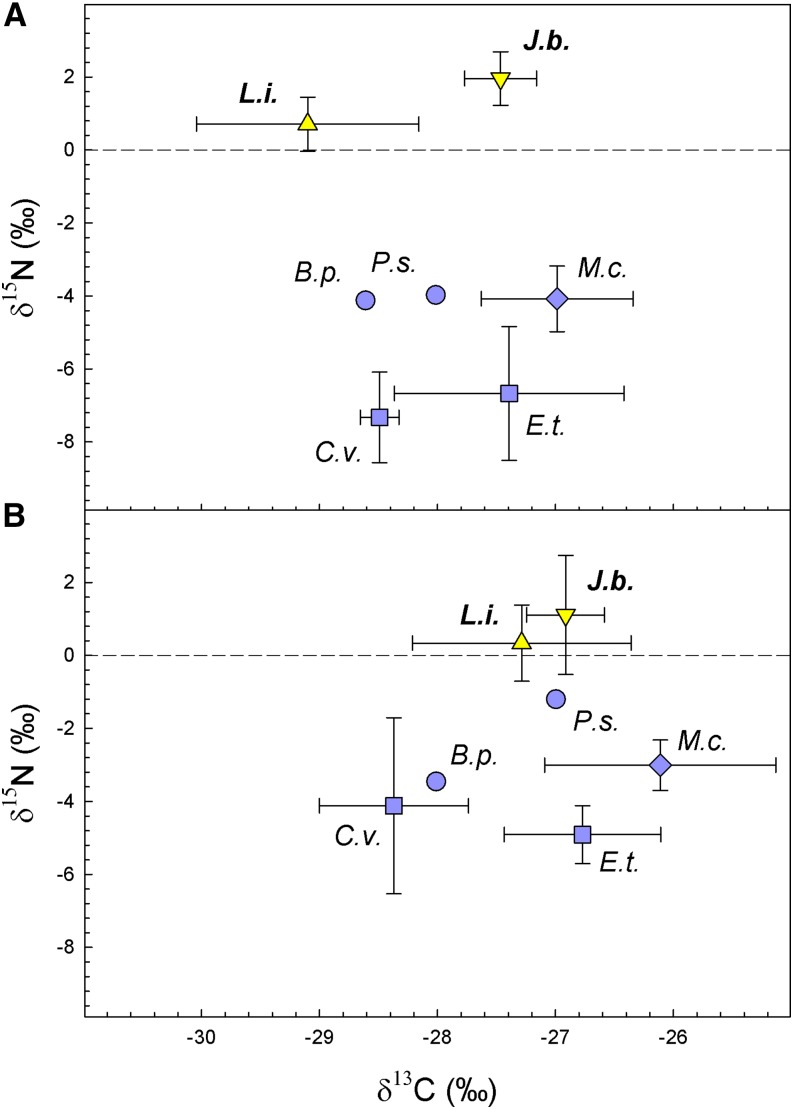
Carbon- and nitrogen-stable isotope natural abundance of *L*. *inundata* (L.i., *n* = 6), *J*. *bulbosus* (J.b., *n* = 6) and surrounding angiosperms (AM: *M*. *caerulea*, M.c., *n* = 6; ectomycorrhizal: *P*. *sylvestris*, P.s., *n* = 1; *B*. *pendula*, B.p., *n* = 1; ericoid mycorrhizal: *C*. *vulgaris*, C.v., *n* = 3, *E*. *tetralix*, E.t., *n* = 6) for leaf (A) and root (B) samples, respectively. Values = means ± sds. One-tailed Kruskal–Wallis test, followed by Dunn’s post hoc procedure, found significant differences (*P >* 0.05) among *L*. *inundata*, *J*. *bulbosus*, and surrounding angiosperms as references in leaf carbon- and nitrogen-stable isotope natural abundance and in root nitrogen-stable isotope natural abundance.

All leaf δ^13^C values from plants collected from the same site as those collected for our isotope tracing experiments ranged between −26.2 and −30.1% and root δ^13^C values between −24.5 and −28.9%, whereas leaf δ^15^N values ranged from 3.3 to −10.0% and root δ^15^N values from 3.1 to −5.9% (Fig. 3). Leaves of the three groups, *L*. *inundata* (*n* = 6), *J*. *bulbosus* (*n* = 6), and reference plants, five species representing three types of mycorrhizal associations, i.e. arbuscular, ericoid, and ectomycorrhizal species (*n* = 17), also collected from the same site as plants used in our isotope tracing experiments, were significantly different in δ^13^C (*H*^2^ = 8.758; *P* = 0.013) and δ^15^N (*H*^2^ = 21.434; *P* < 0.001, Fig. 3A). *L*. *inundata* leaves were significantly depleted in ^13^C compared to *J*. *bulbosus* leaves (*Q* = 2.644, *P* < 0.05) and a significant depletion of *L*. *inundata* leaves compared to reference plant leaves (*Q* = 2.662, *P* < 0.05, Fig. 3A) was found. The *J*. *bulbosus* leaves were not significantly different from reference plants in δ^13^C. No significant difference was discovered for δ^15^N in *L*. *inundata* and *J*. *bulbosus* leaves (*Q* = 1.017, *P* > 0.05), while leaves of both species were significantly enriched in ^15^N compared to the reference plants (*Q* = 2.968, *P* < 0.05; *Q* = 4.205, *P* < 0.05, Fig. 3A). For the roots, only δ^15^N showed significant differences between the three groups under comparison (*F*^2^ = 34.815; *P* < 0.001, Fig. 3B). The *L*. *inundata* and *J*. *bulbosus* roots were not significantly distinguished in δ^15^N; however, roots of both species were significantly enriched in ^15^N compared to reference plant roots (*Q* = 10.109, *P* < 0.001; *Q* = 8.515, *P* < 0.001, Fig. 3B).

### Mucoromycotina FREs of *L*. *inundata* Show Distinctive Cytology

**Figure 4.**
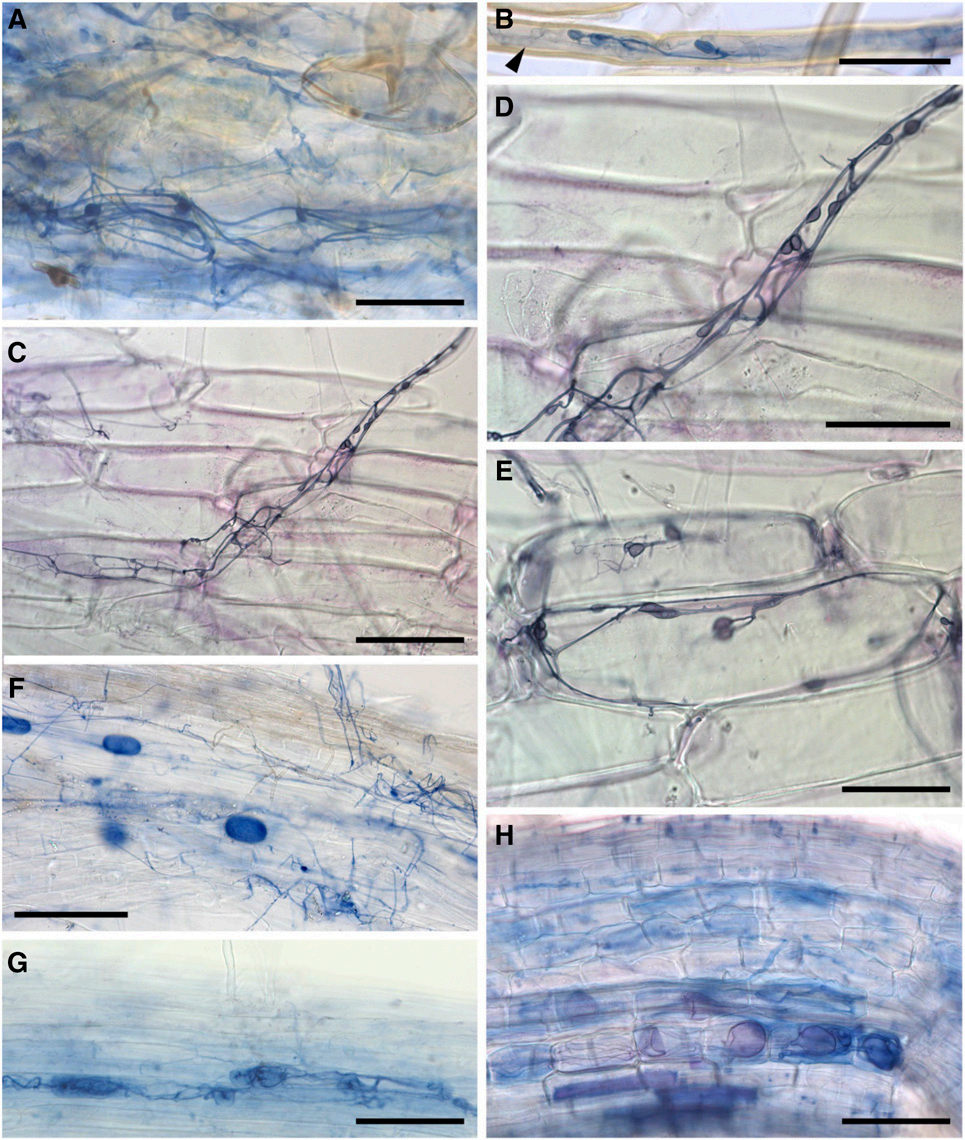
Light micrographs of trypan-blue–stained tissues. A and B, Branching fine hyphae with small swellings/vesicles in thallus cells (A) and rhizoid (B) of the liverwort *F*. *foveolata* (from Thursley Common) colonized by both Mucoromycotina FREs and Glomeromycotina; in (B) also note the coarse hyphae (arrowhead). C to E, Fine hyphae with small swellings/vesicles in the root hairs and root cells of the lycophyte *L*. *inundata* colonized by Mucoromycotina FREs only (field-collected specimens from Thursley Common). F, Fine hyphae with small swellings/vesicles and large vesicles in a root of the grass *H*. *lanatus* (from Lynn Crafnant, Wales) colonized by both Mucoromycotina FREs and Glomeromycotina. G and H, Roots of the grass *M*. *caerulea* (from Thursley Common) colonized by both Mucoromycotina FREs and Glomeromycotina, showing fine hyphae (G) and coarse hyphae with large vesicles (H). Scale bars = 50 μm (A and B, D–F); 100 μm (C, G, and H).

**Figure 5.**
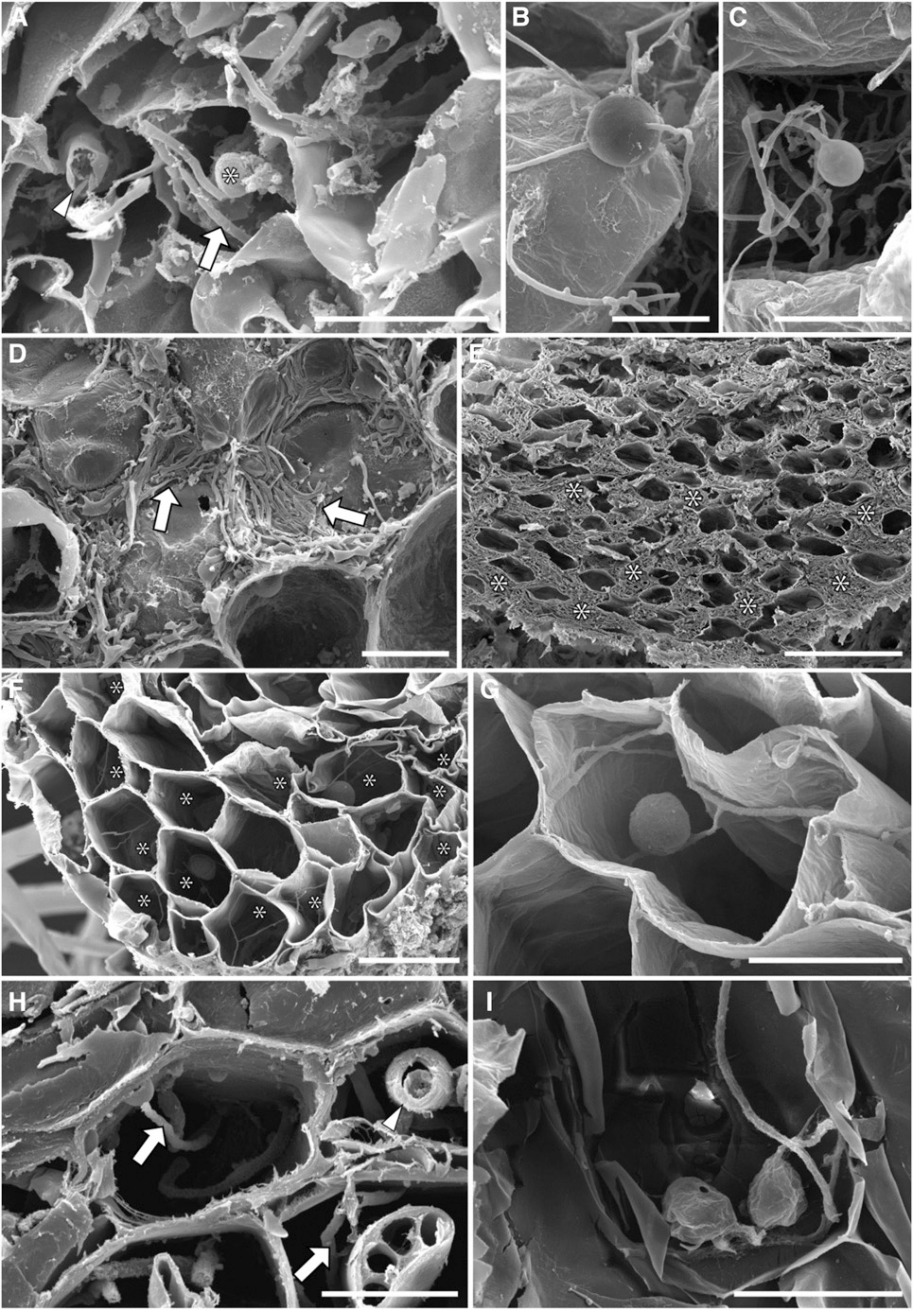
SEM images. A, Fine hyphae (arrows) with a small swelling/vesicle (*) in the thallus cells of *F*. *foveolata* (from Thursley Common); also note the much coarser hyphae (arrowheads). B to G, Fungal colonization in *L*. *inundata*. Intercalary (B) and terminal (C) small swellings/vesicles on fine hyphae in the ventral cell layers of a protocorm (from Thursley Common). Centrally and above this intracellular colonization zone, the fungus becomes exclusively intercellular, as evidenced by the presence of abundant, tightly-appressed hyphae surrounding the central protocorm cells (D, arrows) and eventually completely fills the large, mucilage-filled ICSs present in this zone (E, *). Cross sections of roots of experimental plants; several cells colonized exclusively (*) by branching fine hyphae with small swellings/vesicles (F), enlarged in (G). H and I, Cross sections of roots of *J*. *bulbosus* (from Thursley Common) showing fine (arrows) and coarse (arrowhead) hyphae (H) and a fine hypha with small swellings/vesicles (I). Scale bars = 20 μm (A, D, G, and I), 10 μm (B, C, and H), and 100 μm (E); 50 μm (F).

Trypan blue staining and scanning electron microscopy (SEM) of wild-collected plants (a liverwort, two grasses, and a rush) from the same site as the *L*. *inundata* plants used in our isotope tracer and stable isotope studies (except for the grass *Holcus lanatus*; see Supplemental Table S1), revealed two distinct fungal symbiont morphologies. These consisted of either coarse hyphae (>3-μm diameter) and large vesicles (>20-μm diameter) or fine branching hyphae (<2-μm diameter) with small swellings/vesicles (usually 5–10 but up to 15 μm in diameter; Figs. 4 and 5). Both morphologies were regularly observed, often co-occurring in the same sample, in the gametophyte of the liverwort *Fossombronia foveolata* (Figs. 4, A and B, and 5A; Supplemental Fig. S10A), in the roots of the grasses *H*. *lanatus* (Fig. 4F) and *M*. *caerulea* (Fig. 4, G and H), and the rush *J*. *bulbosus* (Fig. 5, H and I). In the colonized roots of both freshly collected *L*. *inundata* and those incubated in growth chambers, only fine hyphae with small swelling/vesicles were invariably detected (Figs. 4, C–E, and 5, F and G). As in the other plants analyzed, these fine hyphae were aseptate and formed both intercalary and terminal swellings/vesicles but, in contrast to the grasses (Supplemental Fig. S10B), never arbuscules. Similar fungal morphology was also observed in protocorm cells of newly developing sporophytes (Fig. 5, B and C) and in gametophytes of *L*. *inundata* (Supplemental Fig. S11). However, in these early developmental stages, fungal colonization consistently exhibits a distinct zonation: an outer intracellular zone and a more central, strictly intercellular zone (Fig. 5, D and E; Supplemental Fig. S11, B–G). In the intracellular zone, fungal colonization is the same as in the sporophyte roots and consists of fine hyphae with intercalary and terminal swellings/vesicles (Fig. 5, B and C; see also Supplemental Fig. S11I). Unique to the gametophyte generation, in the outermost cortical layers, the fungus also forms tightly wound coils (hyphae up to 2.5 μm in diameter) with larger vesicles (15–20 μm; Supplemental Fig. S11D), as described before in *Lycopodium clavatum* (Schmid and Oberwinkler, 1993). Both gametophyte and early developmental stages of the sporophyte generation develop a conspicuous central system of large, mucilage-filled intercellular spaces (ICSs). In this region, the fungus becomes strictly intercellular (Fig. 5, D and E; Supplemental Fig. S11, E–G). The intercellular hyphae are initially fine and with small swellings/vesicles (Fig. 5D; Supplemental Fig. S11E) as their intracellular counterparts, but soon enlarge and eventually reach diameters in excess of 3 μm (Supplemental Fig. S11F), with no swellings/vesicles present at this stage.

### Mucoromycotina Fungal Symbionts Are Shared by Neighboring Angiosperms

Analysis of *L*. *inundata* plants collected from the same site, at the same time as plants used in the these investigations, confirmed that they were colonized by Mucoromycotina fungi. Glomeromycotina sequences were not detected. Mucoromycotina operational taxonomic units (OTUs) were detected before and after the experiments (Supplemental Table S2); these same OTUs had previously been identified in wild-collected lycophytes from diverse locations (Rimington et al., 2015).

Diverse and shared Mucoromycotina fungi OTUs were detected in wild *L*. *inundata*, liverworts, and angiosperms growing adjacently in the same UK locations (Supplemental Table S2; Supplemental Figs. S2–S8) in the following combinations: *L*. *inundata*, *F*. *foveolata*, *M*. *caerulea*, and *J*. *bulbosus* (Thursley Common, Surrey); *L*. *inundata*, *F*. *foveolata*, and *J*. *bulbosus* (Norfolk); and *F*. *foveolata* and *H*. *lanatus* (Lynn Crafnant, Wales). Mucoromycotina OTUs were also detected in *L*. *inundata* from Studland Heath, Dorset.

## DISCUSSION

Our results show that the symbiosis between *L*. *inundata* and Mucoromycotina FREs is nutritionally mutualistic, with the fungus gaining plant-fixed C and the plant gaining fungal-acquired N and P (Fig. 2; Supplemental Table S3). Cytological analyses of the fungus colonizing the roots of *L*. *inundata* revealed a characteristic morphology consisting invariably of fine, aseptate branching hyphae with terminal and intercalary swellings/vesicles. This morphology matches that described previously in a range of angiosperms colonized by FREs (Orchard et al., 2017a, 2017b) and here in grasses, a rush, and a liverwort, all harboring fungi identified molecularly as Mucoromycotina (Supplemental Fig. S3). Our results provide compelling evidence for Mucoromycotina FREs being shared by plants occupying key nodes in the land plant phylogeny—from early liverworts and vascular lycophytes to the later diverging angiosperms—and demonstrate that this association represents a nutritional mutualism as much in vascular plants as it does in nonvascular plants (Field et al., 2015b, 2016).

Our findings raise important questions regarding the ecology and evolution of mycorrhizal associations and the nature of widespread Mucoromycotina FRE fungal symbioses, chief among which is how have these associations persisted and why are they so widespread today? We can now begin to address this with the demonstration that a vascular plant assimilates relatively large amounts of ^15^N tracer via its Mucoromycotina fungal symbiont when compared to the Mucoromycotina fungal-acquired ^33^P tracer (Fig. 2C), suggesting a potential role for Mucoromycotina FREs in vascular plant nitrogen uptake, complementary to the role of Glomeromycotina AM fungi (Field et al., 2019). This nutritional role could help to explain the persistence of Mucoromycotina FREs across nearly all modern land plant lineages.

### Costs and Benefits of Hosting Mucoromycotina Fungi

Our data demonstrate that *L*. *inundata* transfers carbon to symbiotic Mucoromycotina FRE (Fig. 2, A and B). However, when compared to other vascular plants with Glomeromycotina AM fungal associates in similar experimental systems (Field et al., 2012), it is clear that the relative C “cost” of maintaining Mucoromycotina fungal symbionts in *L*. *inundata* is at least on a par with, if not greater than, that of maintaining Glomeromycotina fungi. It is not entirely clear from our experiments why this might be and it is important to note that the C “cost” of hosting Mucoromycotina FREs has only been tested in one vascular plant species to date and thus represents an important area for future research. It is possible that the carbon-for-nutrient exchange dynamics between plant and Mucoromycotina FREs vary according to plant and fungal identity, in addition to abiotic factors, as it does for Glomeromycotina AM (Field and Pressel, 2018).

Lycophytes represent a critical node in land plant phylogeny, widely considered as a diversification point in the mid-Paleozoic (480–360 million years ago) characterized by the evolution of roots, leaves, stomata, and associated vasculature (Kenrick and Crane, 1997). Given that all the plants sampled grew in close proximity and all follow the C_3_ photosynthetic pathway, the trend for lower δ^13^C values in root tissues versus leaves (Fig. 3) is most likely caused by systematic differences in ^13^C abundance in photosynthetic versus nonphotosynthetic tissues (Gebauer and Schulze, 1991; Cernusak et al., 2009). The depletion of ^13^C observed in the leaves of *L*. *inundata* (Fig. 3A) relative to the other, non-Mucoromycotina associated plants sampled is unlikely to be related to C gains from its Mucoromycotina fungal symbiont (Bago et al., 2000) as one would expect (myco)heterotrophic carbon gains to result in ^13^C enrichment (Press et al., 1987; Schulze et al., 1991; Gebauer and Meyer, 2003); rather, it may indicate that *L*. *inundata* leaves regulate their stomata differently from *J*. *bulbosus* or the reference plants tested, as δ^13^C in tissues of terrestrial plants is, among other factors, driven by the water use efficiency of the plant (Farquhar et al., 1982, 1989).

Alongside increased capacity for regulation of water relations and C capture and fixation, we hypothesize that the increasing size and structural complexity of land plants across the land plant phylogeny and evolutionary time (Field et al., 2012) result in greater plant nutrient demand. Glomeromycotina AM are associated with facilitation of plant P uptake and occur commonly in soils with low P availability (Smith et al., 2015; Albornoz et al., 2016). The amount of ^33^P transferred to *L*. *inundata* plants in our experiments was much less than has previously been recorded for Mucoromycotina-associated liverworts (Field et al., 2016) or for Glomeromycotina-associated ferns and angiosperms (Field et al., 2012) despite the same amount of ^33^P being made available in comparable experimental systems. This suggests Mucoromycotina fungi may not play a critical role in lycophyte P nutrition. Our results contrast with the view that Mucoromycotina FREs enhance plant P uptake, at least in soils with very low P (Ryan and Kirkegaard, 2012; Orchard et al., 2017b). Previous experiments with Mucoromycotina fungi-associated liverworts suggest that in addition to supplying host plants with P, Mucoromycotina fungal associates also play a role in plant N nutrition (Field et al., 2015b, 2016, 2019).

Nitrogen is an essential element for plants that is available in soils in plant-inaccessible organic forms and as plant-accessible inorganic nitrate and ammonium (Krapp, 2015). Our results show that Mucoromycotina FRE symbionts transfer significantly more ^15^N tracer compared to ^33^P (Fig. 2, C and D). Up to 145 times more ^15^N was transferred to *L*. *inundata* (0.3% to 1% of the supplied tracer) than to Haplomitriopsida liverworts in comparable experiments that assimilated between 0.05% and 0.2% of the supplied tracer (Field et al., 2016). Using analysis of natural abundance ^15^N signatures, we show that Mucoromycotina FRE-associated *L*. *inundata* and *J*. *bulbosus* were ^15^N enriched compared to co-occurring reference plants with different mycorrhizal fungal partners (Fig. 3). This ^15^N enrichment could be caused by temporal and spatial variations in N availability and changes in plant N demand over time (Gebauer and Meyer, 2003). However, these factors are unlikely in our case, because all plants sampled from this field collection grew in close spatial proximity and were collected at the same time. Presence of multiple N sources with distinct isotopic values and their utilization by different mycorrhizal associations are known as additional drivers of variations in plant ^15^N isotope abundance (Bidartondo et al., 2004). While this distinction in N isotope abundance between plants with different mycorrhizas is almost or completely lost in conditions of high N availability (Gebauer and Meyer, 2003), it may become prominent under severe N limitation (Schulze et al., 1994). Given that our plants were collected at a heathland field site that was likely N-limited (von Oheimb et al., 2010) despite substantial atmospheric N deposition, the separation of *L*. *inundata* and *J*. *bulbosus* in their natural abundance ^15^N from neighboring plants with other fungal associations supports our hypothesis that plants hosting Mucoromycotina symbionts benefit from fungal-acquired N.

Some Glomeromycotina AM fungi transfer N to their associated hosts (Leigh et al., 2009); however, the ecological relevance of AM-facilitated N uptake is widely debated, in particular the amounts of N transferred to hosts compared to the overall N requirements of the plant (Smith and Smith, 2011). Exclusive plant-Mucoromycotina FRE symbioses seem to be rare, having been reported before only in the earliest-diverging Haplomitriopsida liverworts (Field et al., 2015a, 2015b), while all other plants including other lycophytes (Rimington et al., 2015) that form associations with these fungi, appear able to do so also with Glomeromycotina, often simultaneously (Rimington et al., 2015). It is possible that the large input to *Lycopodiella* N-nutrition and minor contribution to P-nutrition by Mucoromycotina FREs reflect a specialized relationship, particularly pertinent when considering heathland habitats have very low plant-available N. Nevertheless, our present data combined with previous demonstrations of N transfer in liverwort-Mucoromycotina symbioses (Field et al., 2015b, 2016) and emerging evidence that Mucoromycotina FREs, but not Glomeromycotina AM fungi, are able to transfer N to host liverworts from organic sources (Field et al., 2019), all point to a critical role of Mucoromycotina FREs in host plant N nutrition. Indeed, our cytological analyses show that, differently from *Lycopodiella* roots where only fine endophytes were observed (Figs. 4 and 5; Table 1), all other co-occurring plants (*F*. *foveolata*, *J*. *bulbosus*, and *M*. *caerulea*) were also colonized by coarse endophytes with cytology typical of Glomeromycotina (Figs. 4 and 5; Table 1). The finer functional details, in terms of N and P transfer, of this partnership in other vascular plants from a broader range of habitats remain to be established; the challenge here will be to separate the nutritional contributions of Mucoromycotina FREs and Glomeromycotina to host plants that are cocolonized by both fungi (in addition to the contributions made by any mutualistic Ascomycetes and Basidiomycetes), as that seems to be the prevailing condition in vascular plants, especially angiosperms.

**Table 1.** Cytology of colonization and fungal identity of study species compared to relevant examples from the literature referred to in “Discussion.” G = gametophyte generation; S= sporophyte generation.

Plant Group	G/S	Tissue/Location	Colonization	Morphology (Diameter)	Fungus ID	References
Liverworts						
*Treubia*	G	Several ventral cell layers	Intracellular	Coils (0.5–1.5 µm) with “lumps”/swellings (up to 15 μm), arbuscule-like short-side branches on coiled hyphae	M	Duckett et al., 2006; Bidartondo et al., 2011
		Above intracellular zone	Intercellular: large mucilage-filled ICSs	Coarse hyphae 2–3 µm, thick-walled fungal structures		
*Fossombronia*	G	Thallus central strand	Intracellular	Coarse hyphae (2–3 μm); large vesicles (15–30 µm), coils (0.5–1 µm), fine hyphae (0.5–1.5 μm) with small swellings/vesicles (5–10 µm), arbuscules	M&G	This study
Lycophytes						
*Lycopodiella*	G	Outer cortical cell layers	Intracellular	Coils (up to 2.5 µm) with vesicles (15–20 µm)	M	This study
		Several ventral cell layers	Intracellular	Fine hyphae (0.5–1.5 µm) with small swellings/vesicles (5–10 µm)		
		Above intracellular zone	Intercellular: large mucilage-filled ICSs	Coarse hyphae (2–>3 μm)		
	S	Protocorm:	Intracellular	Fine hyphae (0.5–1.5 µm) with small swellings/vesicles (5–10 µm)	M	This study
		Several ventral cell layers central, above intracellular zone	Intercellular: large mucilage-filled ICSs	Coarse hyphae (2–>3 μm)		
	S	Root	Intracellular and intercellular, small ICSs	Fine hyphae (0.5–1.5 µm) with small swellings/vesicles (5–15 µm)	M	Rimington et al., 2015; This study
Angiosperms						
*Holcus*	S	Root	Intracellular and intercellular, small ICSs	Coarse hyphae (>3 µm), large vesicles (20–40 µm), fine hyphae (0.5–1.5 µm) with small vesicles (5–10 µm), arbuscules/arbuscule-like structures	M&G	This study
*Molinia*	S	Root	Intracellular and intercellular, small ICSs	Coarse hyphae (>3 µm), large vesicles (20–40 µm), fine hyphae (0.5–1.5 µm) with small vesicles/swellings (5–10 µm), arbuscules/arbuscule-like structures	M&G	This study
*Juncus*	S	Root	intracellular and intercellular, small ICSs	Coarse hyphae (>3 µm), large vesicles (20–40 µm), fine hyphae (0.5–1.5 µm) with small vesicles (5–10 µm), arbuscules/arbuscule-like structures	M&G	This study
*Trifolium*	S	Root	intracellular and intercellular, small ICSs	Coarse hyphae (>3 μm), large vesicles (>30 μm) fine hyphae (>1.5 μm), intercalary and terminal vesicles/swellings (5–10 μm) and arbuscules/arbuscule-like structures	M&G	Orchard et al., 2017a
Fossils						
*Horneophyton*	S	Aerial axes, cortical cells	intracellular	coarse hyphae (>3 μm), large vesicles (up to 50 μm), arbuscule-like structures	G	Strullu-Derrien et al., 2014
		Corm	intracellular and intercellular	intracellular coils, intercellular coarse hyphae (11–13 μm), thick-walled fungal structures	M	
*Nothia*	S	Aerial and prostrate axes	intercellular and intracellular	coarse hyphae (up to 15 μm) and intercellular vesicles (>50 μm)	Unidentified	Krings et al., 2007

### Mucoromycotina FREs

Mucoromycotina fungi within Endogonales colonizing the gametophytes of liverworts (*F*. *foveolata*) and lycophytes (*L*. *inundata*), the sporophytic protocorms and roots of lycophytes (*L*. *inundata*), and the roots of angiosperms (*J*. *bulbosus*, *M*. *caerulea*, and *H*. *lanatus*), all display the same characteristic morphology attributed previously to FREs (Orchard et al., 2017b; Walker et al., 2018). This contrasts with that typical of Glomeromycotina AM fungal associations, consisting of coarse hyphae (>3-μm diameter) and larger vesicles, which we observed in *Fossombronia*, *Juncus*, *Molinia*, and *Holcus* but not in *L*. *inundata* (Table 1). These observations, together with the molecular identification of Mucoromycotina clades shared by these phylogenetically distant plant lineages presented here, support previous suggestions that vascular plants’ FREs are closely related to the Mucoromycotina mycorrhizal-like symbionts of nonvascular plants (Rimington et al., 2016). Here, we show that the same Mucoromycotina FREs have the capacity to be nutritionally mutualistic across different land plant phyla.

Our demonstration of an extensive intercellular phase of fungal colonization in the gametophytes and protocorms of *L*. *inundata* is in line with other lycophytes (Schmid and Oberwinkler, 1993; Rimington et al., 2015) and strongly recalls the gametophytes of the Haplomitriopsida liverwort *Treubia* (Duckett et al., 2006) and several hornworts (Desirò et al., 2013), all of which have also been shown to associate with Mucoromycotina fungi (Bidartondo et al., 2011; Desirò et al., 2013). Differently from their fine intracellular counterparts, intercellular hyphae become swollen, eventually reaching >3 μm in diameter. Tightly wound hyphal coils up to 2.5 µm in diameter with somewhat larger terminal vesicles (up to 20 μm in diameter) are also prominent in the outer cortical layers of *L*. *inundata* gametophytes but were not observed in either protocorms or roots. Thus, Mucoromycotina FREs display considerable phenotypic plasticity in their interactions with diverse lineages of land plants that appears to relate to the developmental stage of the host and whether it produces an extensive network of mucilage-filled ICSs. The putative occurrence of Mucoromycotina FREs in early land plants and their presence in both extant early and later diverging plant lineages now point to a prominent role of these fungi, not only in plant terrestrialization (Field et al., 2015a), but also in current ecosystem functioning. Indeed, Mucoromycotina FREs have been shown to occur worldwide across many ecosystems, particularly in the roots of crop and pasture species where colonization levels may be high, even as dense as the biomass of coarse Glomeromycotina arbuscular mycorrhizal fungi (Orchard et al., 2017b).

## CONCLUSION

### More Ammunition for the Mycorrhizal Revolution

Our findings provide conclusive evidence that Mucoromycotina FREs form nutritional mutualisms not only with nonvascular liverworts (Field et al., 2015b, 2016), but also with a vascular plant. We have found that the Mucoromycotina FRE associates of *L*. *inundata* receive up to 189 times more photosynthesis-derived C from the plant than the Mucoromycotina fungal associates of nonvascular plants (Field et al., 2016). In return, *L*. *inundata* hosts receive a relatively large amount of N from their Mucoromycotina FRE partners—∼145 times more than nonvascular plants receive from their Mucoromycotina fungal symbionts (Field et al., 2016). Together with our discovery that the same Mucoromycotina fungal symbionts are shared with neighboring grasses, rushes and liverworts, and recent findings of functional complementarity between Mucoromycotina FREs and Glomeromycotina AM (Field et al., 2019), our findings point toward a unique physiological niche for the persistence of Mucoromycotina fungi, both in single and dual colonizations with Glomeromycotina AM.

## MATERIALS AND METHODS

### Plant Material and Growth Conditions

*Lycopodiella inundata*, neighboring angiosperms (the grasses *Holcus lanatus*, *Molinia caerulea*, and the rush *Juncus bulbosus*), and a liverwort (*Fossombronia foveolata*) were collected from Thursley National Nature Reserve, Surrey, United Kingdom (SU 90081 39754), a heathland site, in June 2017. The *L*. *inundata* plants were planted directly into pots (90-mm diameter × 85-mm depth) containing a homogenous mixture of acid-washed silica sand and 5% pot volume compost (No. 2; Petersfield) to aid water-retention properties of the substrate and to provide minimal nutrients. Soil surrounding plant roots was left intact to prevent damage to the roots and to act as a natural inoculum, including symbiotic fungi and associated microorganisms. Pots were weeded regularly to remove other plant species. All plants used throughout this investigation were collected from the wild. Each microcosm was a homogenized mixture of acid-washed sand and soil collected from around the plant roots from the collection site. All plants used in this investigation (isotope tracing, cytology, and stable isotope studies) were collected from Thursley Nature Reserve, with additional plants from three other United Kingdom field sites (Supplemental Table S1), used for additional cytological and molecular analyses.

Based on the methods of Field et al. (2012, 2015a), three windowed cylindrical plastic cores covered in 10-μm nylon mesh (Supplemental Fig. S1) were inserted into the substrate within each experimental pot. Two of the cores were filled with the same substrate as the bulk soil within the pots, comprising a homogenous mixture of acid-washed silica sand and compost (No. 2; Petersfield), together making up 95% of the core volume, native soil gathered from around the roots of wild plants to ensure cores contained the same microbial communities as in bulk soil (4% core volume), and fine-ground tertiary basalt (1% core volume) to act as fungal bait. The third core was filled with glass wool to allow below-ground gas sampling throughout the ^14^C-labeling period to monitor soil community respiration. Plants were watered every other day with no additional applications of nutrient solutions. Microcosms shared a common drip-tray within each cabinet throughout the acclimation period that ensured a common pool of rhizospheric microorganisms in each microcosm.

A total of 20 *L*. *inundata* microcosms were maintained in controlled environment chambers (model no. Micro Clima 1200; Snijders Labs) with a light cycle of 16-h daytime (20°C and 70% humidity) and 8-h night-time (at 15°C and 70% humidity). Day-time photosynthetically active radiation (PAR), supplied by LED lighting, was 225 μmol photons m^−2^ s^−1^. Atmospheric CO_2_ concentrations were set at 440 μL L^−1^. Atmospheric [CO_2_] was monitored using a sensor system (Vaisala), maintained through addition of gaseous CO_2_. All pots were rotated within cabinets to control for cabinet and block effects. Plants were acclimated to chamber/growth regimes for four weeks to allow establishment of mycelial networks within pots and confirmed by hyphal extraction from soil and staining with trypan blue (Brundrett et al., 1996). Additionally, roots were stained with acidified ink for the presence of fungi, based on the methods of Brundrett et al. (1996).

### Molecular Identification of Fungal Symbionts and Phylogenetic Analysis

All plants (Supplemental Table S1) were processed for molecular analyses within 1 week of collection. Genomic DNA extraction and purification from all specimens and subsequent amplification, cloning, and Sanger sequencing were performed according to methods from Rimington et al. (2015). The fungal 18S rRNA gene was targeted using the broad specificity fungal primer set NS1/EF3 and a semi-nested approach with Mucoromycotina- and Glomeromycotina-specific primers described in Desirò et al. (2013) for the experimental *L*. *inundata* plants and all other field-collected plant material. Resulting partial 18S rRNA sequences ∼400–700 bp were edited and preliminarily identified with the tool “BLAST” (https://blast.ncbi.nlm.nih.gov/Blast.cgi) using the software “Geneious 8.1.7” (Kearse et al., 2012). Chimeric sequences were detected using the “UCHIME2” algorithm (Edgar, 2016) in conjunction with the most recent nonredundant small subunit SILVA database (small subunit Ref NR 132, December 2017; www.arb-silva.de). Sequences identified as Mucoromycotina sp were aligned with the tool “MAFFT” before removing unreliable columns using the default settings in the software “GUIDANCE2” (http://guidance.tau.ac.il). The best-fit nucleotide model for phylogenetic analysis was calculated using “Smart Model Selection” (Kumar et al., 2016). Maximum likelihood with 1,000 replicates was performed using the software “PhyML 3.0” (Guindon and Gascuel, 2003). Bayesian inference analysis was conducted in the software “MrBayes 3.2.6” (Ronquist and Huelsenbeck, 2003) with four Markov-chain Monte Carlo strands and 10^6^ generations. Consensus trees were produced after excluding an initial burn-in of 25% of the samples (Supplemental Figs. S3–S9). Representative DNA sequences were deposited in the GenBank (see “Accession Numbers”).

### Cytological Analyses

*L*. *inundata* gametophytes (*n* = 15), young sporophytes (protocorms; *n* = 15), and roots of mature plants (both wild [*n* = 20] and experimental [*n* = 20]), roots of angiosperms (*H*. *lanatus*, *M*. *caerulea*, and *J*. *bulbosus*; *n* = 30 for each species), and liverwort gametophytes (*F*. *foveolata; n* = 30) were either stained with trypan blue (Brundrett et al., 1996) and photographed under an Axioscope (Zeiss) equipped with a digital camera (MRS Systems), or processed for SEM within 48 h of collection. For SEM, we followed the protocol of Duckett et al. (2006); see Supplemental Materials and Methods).

### Quantification of C, ^33^P, and ^15^N Fluxes between Lycophytes and Fungi

After the 4-week acclimation period, microcosms were moved to individual drip-trays immediately before isotope labeling to avoid cross-contamination of the isotope tracers. One-hundred microliters of an aqueous mixture of ^33^P-labeled orthophosphate (specific activity 111 TBq mmol^−1^, 0.3 ng ^33^P added; Hartmann Analytics) and ^15^N-ammonium chloride (1 mg ml^−1^; 0.1 mg ^15^N added; Sigma-Aldrich) was introduced into one of the soil-filled mesh cores in each pot through the installed capillary tube (Supplemental Fig. S2A). In half (*n* = 10) of the pots, cores containing isotope tracers were left static to preserve direct hyphal connections with the lycophytes. Fungal access to isotope tracers was limited in the remaining half (*n* = 10) of the pots by rotating isotope tracer-containing cores through 90°, thereby severing the hyphal connections between the plants and core soil. These were rotated every second day thereafter, thus providing a control treatment that allows us to distinguish between fungal and microbial contributions to tracer uptake by plants. Assimilation of ^33^P tracer into above-ground plant material was monitored daily using a hand-held Geiger counter held over the plant material.

At detection of peak activity in above-ground plant tissues (21 d after the addition of the ^33^P and ^15^N tracers), the tops of ^33^P- and ^15^N-labeled cores were sealed with plastic caps and anhydrous lanolin and the glass wool cores were sealed with rubber septa (SubaSeal; Sigma-Aldrich). Before cabinet lights were turned on at 8 am, each pot was sealed into a 3.5-L, gas-tight labeling chamber and 2 mL of 10% (w/v) lactic acid was added to 30 μL of NaH^14^CO_3_ (specific activity 1.621 GBq/mmol^−1^; Hartmann Analytics), releasing a 1.1-MBq pulse of ^14^CO_2_ gas into the headspace of the labeling chamber (Supplemental Fig. S2B). Pots were maintained under growth cabinet conditions, and 1 mL of headspace gas was sampled after 1 h and every 1.5 h thereafter. Below-ground respiration was monitored via gas sampling from within the glass-wool–filled core after 1 h and every 1.5 h thereafter for ∼16 h.

### Plant Harvest and Sample Analyses

Upon detection of maximum below-ground flux of ^14^C, ∼16 h after the release of the ^14^CO_2_ pulse, each microcosm compartment (i.e. plant material and soil) was separated, freeze-dried, weighed, and homogenized. The ^33^P activity in plant and soil samples was quantified by digesting in concentrated H_2_SO_4_ (Supplemental Materials and Methods) and liquid scintillation (Tricarb 3100TR liquid scintillation analyzer; Isotech). The quantity of ^33^P tracer that was transferred to the plant by its fungal partner was then calculated using previously published equations (Cameron et al., 2007; see Supplemental Materials and Methods). To determine total symbiotic fungal-acquired ^33^P transferred to *L*. *inundata*, the mean ^33^P content of plants that did not have access to the tracer because cores into which the ^33^P was introduced were rotated, was subtracted from the total ^33^P in each plant that did have access to the isotopes within the core via intact fungal hyphal connections (i.e. static cores). This calculation controls for diffusion of isotopes and microbial nutrient cycling in pots, ensuring only ^33^P gained by the plant via intact fungal hyphal connections is accounted for and therefore serves as a conservative measure of the minimum fungal transfer of tracer to the plant.

Between 2 and 4 mg of freeze-dried, homogenized plant tissue was weighed into 6 × 4 mm^2^ tin capsules (Sercon) and ^15^N abundance was determined using a continuous flow infrared mass spectormetry (IRMS; model no. PDZ 2020 IRMS; Sercon). Air was used as the reference standard, and the IRMS detector was regularly calibrated to commercially available reference gases. The ^15^N transferred from fungus to plant was then calculated using equations published in Field et al. (2016); see Supplemental Materials and Methods). In a similar manner as for the ^33^P tracer, the mean of the total ^15^N in plants without access to the isotope because of broken hyphal connections between plant and core contents was subtracted from the total ^15^N in each plant with intact hyphal connections to the mesh-covered core to give fungal-acquired ^15^N. Again, this provides a conservative measure of ^15^N transfer from fungus to plant as it ensures only ^15^N gained by the plant via intact fungal hyphal connections is accounted for.

The ^14^C activity of plant and soil samples was quantified through sample oxidation (307 Packard Sample Oxidizer, Isotech) followed by liquid scintillation. Total C (^12^C + ^14^C) fixed by the plant and transferred to the fungal network was calculated as a function of the total volume and CO_2_ content of the labeling chamber and the proportion of the supplied ^14^CO_2_ label fixed by plants (see Supplemental Materials and Methods). The difference in total C between the values obtained for static and rotated core contents in each pot is considered equivalent to the total C transferred from plant to symbiotic fungus within the soil core for that microcosm, noting that a small proportion will be lost through soil microbial respiration. The total C budget for each experimental pot was calculated using equations from Cameron et al. (2006); see Supplemental Materials and Methods). Total percent allocation of plant-fixed C to extraradical symbiotic fungal hyphae was calculated by subtracting the activity (in becquerels) of rotated core samples from that detected in static core samples in each pot, dividing this by the sum of activity detected in all components of each microcosm, then multiplying it by 100.

### Stable Isotope Signatures of Neighboring Plants

*L*. *inundata* and *J*. *bulbosus* were collected from Thursley National Nature Reserve, Surrey, together with co-occurring reference plants from six 1-m^2^ plots in May 2018, following the sampling scheme of Gebauer and Meyer (2003). Five plant species representing three different types of mycorrhizal associations served as reference plants: two ericoid mycorrhizal species (*Erica tetralix*, collected on six plots; *Calluna vulgaris*, collected on three plots), two ectomycorrhizal species (*Pinus sylvestris* and *Betula pendula* seedlings, both from one plot), and one arbuscular mycorrhizal species (*M*. *caerulea* from six plots). Relative carbon and nitrogen isotope natural abundances of dried and ground leaf and root samples were measured in a dual element analysis mode with a model no. 1108 elemental analyzer (Carlo Erba Instruments) coupled to a DELTA S Continuous Flow Isotope Ratio Mass Spectrometer (using a Finnigan MAT; Thermo Fisher Scientific) via a ConFlo III open-split interface (Thermo Fisher Scientific), as described in Bidartondo et al. (2004). Relative isotope abundances (δ values) were calculated, calibrated, and checked for accuracy using methods detailed in Supplemental Materials and Methods.

### Statistics

Isotope tracing data were checked for normality and differences between plant assimilation of ^33^P and ^15^N were tested using Student’s *t* test with the software “SPSS v24” (IBM). Mean values are displayed in figures with se. Stable isotope patterns between the groups of *L*. *inundata* (*n* = 6), *J*. *bulbosus* (*n* = 6), and surrounding angiosperms (*n* = 17) were tested for normality and equal variance. A one-tailed Kruskal–Wallis test (*Q*) was applied for nonparametric data followed by Dunn’s post hoc procedure, while one-way ANOVA (*F*) was applied for parametric data followed by the Tukey post hoc procedure (*q*). Mean values are displayed in figures with sd.

### Accession Numbers

Representative DNA sequences were deposited in the GenBank/EMBL data libraries under accession numbers MK673773–MK673803.

### Supplemental Data

The following supplemental materials are available.

**Supplemental Figure S1.** Schematic diagram of mesh-covered core showing dimensions of window (not drawn to scale).
**Supplemental Figure S2.** Schematic diagrams of experimental microcosms showing N and P, and C isotope tracing.
**Supplemental Figure S3.** Phylogenetic relationships of Mucoromycotina OTUs associated with *L*. *inundata* grown for isotope tracing and cytology experiments in controlled environment growth chambers.
**Supplemental Figure S4.** An overview of phylogenetic relationships of Mucoromycotina OTUs associated with bryophytes, lycophytes, and angiosperms from various UK locations based on partial 18S gene sequences.
**Supplemental Figure S5.** Phylogenetic relationships of partial 18S DNA sequences classified as OTU 2, corresponding to “group A” in Desirò et al. (2013).
**Supplemental Figure S6.** Phylogenetic relationships of partial 18S DNA sequences classified as OTU 1, 3, and 4.
**Supplemental Figure S7.** Phylogenetic relationships of partial 18S DNA sequences clustering within OTU 5.
**Supplemental Figure S8.** Phylogenetic relationships of partial 18S DNA sequences classified as OTU 6, corresponding to “group B” in Desirò et al. (2013).
**Supplemental Figure S9.** Phylogenetic relationships of partial 18S DNA sequences clustering with OTU 7, corresponding to “group I” in Desirò et al. (2013).
**Supplemental Figure S10.** Micrographs showing morphology of fungal colonization in *F*. *foveolata* and *H*. *lanatus*.
**Supplemental Figure S11.** SEM images and light micrograph of toluidine blue stained semi-thin sections. Gametophyte morphologies in *L*. *inundata* (from Thursley Common).
**Supplemental Table S1.** Samples of lycophytes, liverworts, and angiosperms analyzed with their origin.
**Supplemental Table S2.** A summary of Mucoromycotina OTUs associated with liverworts, lycophytes, and angiosperms at four UK sites.
**Supplemental Table S3.** A summary of the amounts of C, ^15^N, and ^33^P detected in static and rotated core of microcosms used during carbon-for-nutrient experiments between *L*. *inundata* and Mucoromycotina FRE fungi.
**Supplemental Materials and Methods.** Detailed methods for mesh-covered core construction, calculation of carbon and nutrient fluxes between symbionts, molecular methods for fungal identification and cytological analyses of resin-embedded plant material.
